# Comparative study of extracellular vesicles derived from mesenchymal stem cells and brain endothelial cells attenuating blood–brain barrier permeability via regulating Caveolin-1-dependent ZO-1 and Claudin-5 endocytosis in acute ischemic stroke

**DOI:** 10.1186/s12951-023-01828-z

**Published:** 2023-02-28

**Authors:** Yiyang Li, Bowen Liu, Tingting Zhao, Xingping Quan, Yan Han, Yaxin Cheng, Yanling Chen, Xu Shen, Ying Zheng, Yonghua Zhao

**Affiliations:** 1 Institute of Chinese Medical Sciences, State Key Laboratory of Quality Research in Chinese Medicine, University of Macau, Taipa, Macau SAR China; 2 Key Laboratory of Neuropharmacology and Translational Medicine of Zhejiang Province, School of Pharmaceutical Sciences, Zhejiang Chinese Medical University, Hangzhou, China; 3 Faculty of Chinese Medicine, Macau University of Science and Technology, Taipa, Macao SAR China; 4 Department of Pathophysiology, Zhuhai Campus of Zunyi Medical University, Zhuhai, Guangdong China; 5 Jiangsu Key Laboratory for Pharmacology and Safety Evaluation of Chinese Materia Medica and State Key Laboratory Cultivation Base for TCM Quality and Efficacy, Nanjing University of Chinese Medicine, Nanjing, China; 6 Department of Pharmaceutical Sciences, Faculty of Health Sciences, University of Macau, Taipa, Macau SAR China

**Keywords:** Blood–brain barrier, Caveolin-1, Endocytosis, Extracellular vesicles, Ischemic stroke, Tight junction proteins

## Abstract

**Background:**

Blood–brain barrier (BBB) disruption is a major adverse event after ischemic stroke (IS). Caveolin-1 (Cav-1), a scaffolding protein, played multiple roles in BBB permeability after IS, while the pros and cons of Cav-1 on BBB permeability remain controversial. Numerous studies revealed that extracellular vesicles (EVs), especially stem cells derived EVs, exerted therapeutic efficacy on IS; however, the mechanisms of BBB permeability needed to be clearly illustrated. Herein, we compared the protective efficacy on BBB integrity between bone marrow mesenchymal stem cells derived extracellular vesicles (BMSC-EVs) and EVs from brain endothelial cells (BEC-EVs) after acute IS and investigated whether the mechanism was associated with EVs antagonizing Cav-1-dependent tight junction proteins endocytosis.

**Methods:**

BMSC-EVs and BEC-EVs were isolated and characterized by nanoparticle tracking analysis, western blotting, and transmission electron microscope. Oxygen and glucose deprivation (OGD) treated b. End3 cells were utilized to evaluate brain endothelial cell leakage. CCK-8 and TRITC-dextran leakage assays were used to measure cell viability and transwell monolayer permeability. Permanent middle cerebral artery occlusion (pMCAo) model was established, and EVs were intravenously administered in rats. Animal neurological function tests were applied, and microvessels were isolated from the ischemic cortex. BBB leakage and tight junction proteins were analyzed by Evans Blue (EB) staining and western blotting, respectively. Co-IP assay and Cav-1 siRNA/pcDNA 3.1 vector transfection were employed to verify the endocytosis efficacy of Cav-1 on tight junction proteins.

**Results:**

Both kinds of EVs exerted similar efficacies in reducing the cerebral infarction volume and BBB leakage and enhancing the expressions of ZO-1 and Claudin-5 after 24 h pMCAo in rats. At the same time, BMSC-EVs were outstanding in ameliorating neurological function. Simultaneously, both EVs treatments suppressed the highly expressed Cav-1 in OGD-exposed b. End3 cells and ischemic cerebral microvessels, and this efficacy was more prominent after BMSC-EVs administration. Cav-1 knockdown reduced OGD-treated b. End3 cells monolayer permeability and recovered ZO-1 and Claudin-5 expressions, whereas Cav-1 overexpression aggravated permeability and enhanced the colocalization of Cav-1 with ZO-1 and Claudin-5. Furthermore, Cav-1 overexpression partly reversed the lower cell leakage by BMSC-EVs and BEC-EVs administrations in OGD-treated b. End3 cells.

**Conclusions:**

Our results demonstrated that Cav-1 aggravated BBB permeability in acute ischemic stroke, and BMSC-EVs exerted similar antagonistic efficacy to BEC-EVs on Cav-1-dependent ZO-1 and Claudin-5 endocytosis. BMSC-EVs treatment was superior in Cav-1 suppression and neurological function amelioration.

**Graphical Abstract:**

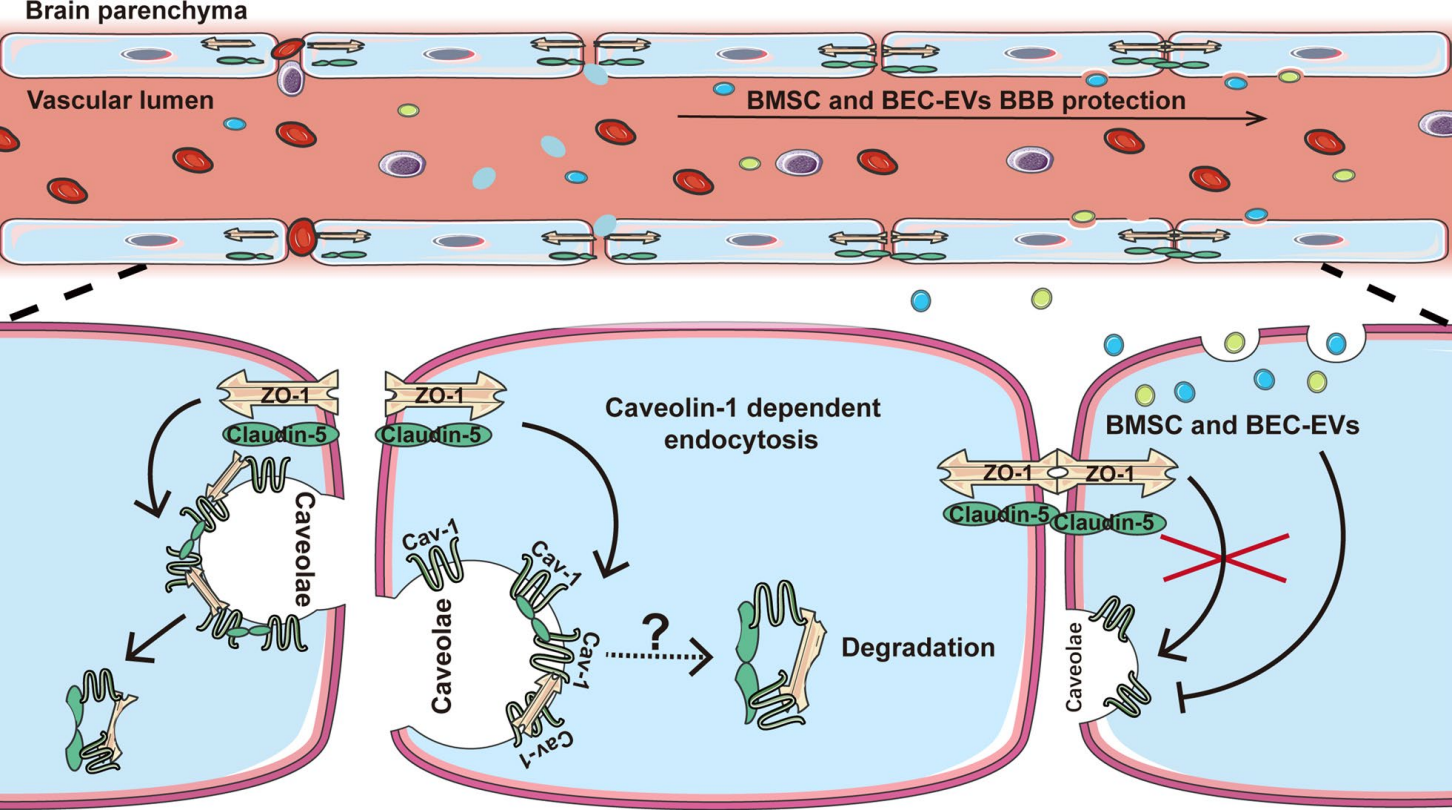

**Supplementary Information:**

The online version contains supplementary material available at 10.1186/s12951-023-01828-z.

## Introduction

As a severe cerebral vascular disease that accounts for nearly 10% of mortality and 5% of disability globally, stroke has brought miserable medical and economic burdens to patients and society [1]. Annually, about 795,000 people suffered from stroke based on the data from the National Heart, Lung, and Blood Institute (NHLBI) of the United States, which led to $52.8 billion in disease-related costs between 2017 and 2018. Among all types of stroke occurrences, about 87% are categorized as ischemic stroke (IS) [2]. Effective intervention measures are still limited for IS treatment, and one of the therapeutics is intra-arterial or intravenous thrombolysis by administration of tissue plasminogen activator (t-PA). However, due to the narrow therapeutic time window and individual contraindications, only < 6.5% of patients benefited from the therapy in the USA, and hemorrhagic transformation is difficult to avoid [3–5]. Another therapeutic measure is mechanical thrombectomy. As an invasive endovascular approach, it is suitable for patients with large artery occlusion, but it also produces high risks, e.g., vascular injury, intracerebral hemorrhage, and new territory occlusion [6]. Moreover, although 95% of the published preclinical studies showed neuroprotective drugs had positive efficacies in animal IS models from 1990–2018, few achieved satisfactory outcomes in clinical Phase III trials [7]. Therefore, exploiting novel therapeutic approaches is essential.

The blood–brain barrier (BBB) acts as a dynamic “doorkeeper” to mediate the liquid and substance exchange for maintaining the homeostasis between brain parenchyma and peripheral circulation [8]. However, once the function and structure of the neurovascular unit (NVU) are destroyed, it renders plenty of peripheral substances and immune cells to enter the brain parenchyma and eventually aggravates neurological functional deficit [9]. During pathological progression, transcytosis and paracellular barrier opening mediated by translocation and degradation of tight junction (TJ) proteins account for two significant modes of BBB permeability [9]. Caveolin-1 (Cav-1), as a caveolae scaffolding protein, predominated in the transcytosis process instead of the paracellular barrier in the early stage of ischemia/reperfusion (IR) [10]. However, the relationship between Cav-1 and TJ proteins is still controversial [11]. Studies indicated focal cerebral IR enhanced the production of nitric oxide (NO), resulting in the loss of Cav-1 and eventually contributed to matrix metalloproteinases (MMPs) activation for the degradation of TJ proteins [12]. Besides, the redistribution of Claudin-5 in cerebral microvessels aggravated BBB permeability, and the mechanism was related to the high expression of Cav-1 in ischemic brain endothelial cells (BECs) after two-hour middle cerebral artery occlusion (MCAo) in rats [13]. Although the paradoxical phenomenon was attributed to different experimental models and the discrepancy of observation time points after IS [14], the efficacy of Cav-1 on TJ protein regulation still awaits to be illustrated in acute IS.

Extracellular vesicles (EVs) are natural phospholipid bilayer tiny vesicles secreted by almost all kinds of cells. Their size and characteristics enable them to penetrate BBB easily [15, 16]. In IS treatment, EVs have presented the potential to facilitate neuro-angiogenesis, regulate immune response and inflammation, and ameliorate neurological function [17]. Since cell transplantation therapy became popular in recent studies, stem cell therapy has been widely proven to bring magnificent benefits in IS recovery and has been conducted in clinical trials [18], however, potential side effects, including uncertainty of biodistribution and uncontrollable cell differentiation, limit cell transplantation therapy development. Compared with bone marrow mesenchymal stem cells (BMSC) transplantation for IS treatment, stem cells originated EVs exert similar efficacy and partly avoid adverse reactions such as undesirable differentiation, vascular embolism, and seizure [19]. Transplanting EVs from optimal cell sources should be a promising approach for IS treatment. NVU cells-derived BEC-EVs exerted satisfied neurological recovery efficacy as previously reported after IS [20–22]. Similarly, BMSCs derived EVs treatment more widely mediated multiple pathways for IS recovery [23–25]. Nevertheless, few studies report their efficacies on BBB disruption in acute ischemic stroke, and virtually seldom research demonstrates the outcome discrepancy among EV treatments from different cell origins.

In this study, we isolated EVs from cultured BMSCs and BECs and illustrated their efficacies on BBB integrity and neurological functional improvement in the rat permanent MCAO model. Simultaneously, we firstly defined the effectiveness of BMSC-EVs against Cav-1 expression was superior to that of BEC-EVs and described the colocalization of Cav-1 with TJ proteins (ZO-1 and Claudin-5) in BECs under hypoxic conditions. Also, our results suggested that the therapeutic mechanism of EVs from two sources of cells on BBB permeability was characterized by the regulation of Cav-1- dependent ZO-1 and Claudin-5 endocytosis.

## Materials and methods

### Isolation of EVs

EVs derived from BMSCs and BECs were isolated by ultrafiltration and Exo-Prep kit (HansaBioMed Life Sciences) according to the manufacturer’s protocol. Briefly, BMSCs and BECs were seeded into 75 cm^3^ flasks and cultured to 50–60% confluence. Then, EVs-deprived fetal bovine serum (EVs-free FBS) was prepared by ultracentrifuge at 100,000 g for 18 h under 4 ℃ (XPN-100, Beckman Coulter) according to a previous report [26] and then was added into culture medium when cells grew to 70–80% confluence. Eventually, cells were allowed to grow for another 24 h. After that, the supernatants were collected, centrifuged under 1000 g for 5 min, filtered by 0.22 μm membrane filters (Millipore), and then concentrated by ultrafiltration spin columns (28932358, Cytiva). Exo-prep reagent was added to the concentrated supernatant and left to stand for 1 h on ice until centrifuged at 10,000 g for another 1 h under 4 ℃. The EVs-enriched precipitation was washed with cold PBS 3 times and centrifuged again at 10,000 g under 4 ℃ for 5 min to fully abandon the supernatant and reagent. The final precipitation, including BEC-EVs or BMSC-EVs, was resuspended in 200 μl PBS and stored at − 80 ℃ refrigerator till further experiments. All samples were filtered again using 0.22 μm membrane filters to ensure sample sterility before administration.

### EVs size distribution, morphology identification, and phagocytosis experiment

The size distribution of BEC-EVs and BMSC-EVs was determined by a nanoparticle tracking analysis (NTA) device (Nanosight-NS500, Malvern Panalytical Ltd), and the morphological images were taken by transmission electron microscope (TEM) under 40 k × magnification (Service provided by Servicebio Inc.). Western blotting was used to identify the surface markers (TSG 101, HSP 70, Alix, CD 9, and CD 63). To verify EVs from two sources of cells were phagocytized by BECs, they were labeled by 5 μM DiI (V22885, Invitrogen) and then washed by PBS 3 times to remove the excess dye, and DiI labeled BEC/BMSC-EVs were then co-cultured with b. End3 cells for 10–120 min under 37 ℃ or 4 ℃. Subsequently, cells were fixed by 4% paraformaldehyde (PFA) and permeabilized by 0.5% Triton X-100. Nucleus was stained by DAPI (C1005, Beyotime) before observation. Images were taken by a confocal microscope (SP8, Leica) under 63 × objective. For negative control, DMEM containing EVs-free FBS was subjected to EVs isolation and DiI dye labeling, the same as EVs samples. The negative control samples were also applied to NTA, TEM, and phagocytosis experiments.

### Cell cultures and OGD treatment

BECs (b. End3 mouse brain endothelial cell line) and rat BMSCs (Purchased from Shanghai Zhong Qiao Xin Zhou Biotechnology Co., Ltd) were cultured in Dulbecco’s modified Eagle’s medium (DMEM) (11965092, Gibco) with 1% penicillin/streptomycin (15070063, Gibco) and 10% fetal bovine serum (FBS) (26140079, Gibco) under 37℃ with 95% O_2_ and 5% CO_2_. Cells within 10 passages were employed in the study. BECs were subjected to 4 or 6 h-oxygen and glucose deprivation (OGD) by a hypoxic chamber to mimic the hypoxic and glucose shortage status. Briefly, the medium of BECs was replaced with glucose-deprived DMEM (11966025, Gibco), and cells were transferred to a hypoxic chamber (MIC-101, Billups-Rothenberg). The oxygen percentage in the chamber was determined by Nuvair O_2_ QuickStick (Nuvair). The ventilation rate of N_2_ was controlled between 10–20 L/min, and the chamber was sealed until the O_2_% ≤ 0.5%, and then BECs were cultured at 37 ℃ for 4 or 6 h.

### Determination of optimal OGD duration and EVs administered dosage

To determine optimal OGD duration and dosage of EVs administration, b. End3 cells at the density of 1 × 10^4^ cells/well were seeded into 96 well plates and cultured overnight at 37 ℃ with 5% CO_2_, and then 4 or 6-h-OGD was employed. After that, the medium containing 10% CCK-8 solution (C0037, Beyotime) was replaced in each well. BECs were cultured for another 2 h until the absorbance of each well was read by a microplate reader (M5, SpectraMax) with the excitation wavelength at 450 nm. OD value of each well was read, normalized, and presented as ratio vs. Control group (set as 1).

For suitable OGD duration assessment, cell viability in groups of Control, OGD 4 h, and OGD 6 h was evaluated. And for optimal EVs dosage appraisal, the particle number of isolated EVs was determined by NTA. BECs were divided into 8 groups as follows: Control, OGD, OGD + low dosage of BEC-EVs (~ 5 × 10^9^ BEC-EVs), OGD + middle dosage of BEC-EVs (~ 1 × 10^10^ BEC-EVs), OGD + high dosage of BEC-EVs (~ 2 × 10^10^ BEC-EVs), and OGD + low dosage of BMSC-EVs (~ 5 × 10^9^ BMSC-EVs), OGD + middle dosage of BMSC-EVs (~ 1 × 10^10^ BMSC-EVs), OGD + high dosage of BMSC-EVs (~ 2 × 10^10^ BMSC-EVs).

### Permanent MCAo model establishment, EVs administration, and animal grouping

The animal experiment protocol was approved by the Ethics Committee of the University of Macau (Ethics number: UMARE-035–2020), and animal benefits were ensured according to the Guide for the Care and Use of Laboratory Animals (8th edition, Washington, DC: The National Academies Press, 2011). Healthy male Sprague–Dawley (SD) rats (weighing 250-280 g, ~ 8 weeks) were anesthetized by 1.5% (w/v) sodium pentobarbital intraperitoneal injection (30 mg/kg) before they suffered from surgical procedure. A longitudinal midline incision in the neck was made at 2–3 cm below the incisor tooth, and common carotid, internal carotid, and external carotid arteries separation was performed. Then, a micro-cut was made at the common carotid artery, and 0.25 mm-diameter monofilament thread with a silicone top was inserted via the cut and advanced 1.6–1.8 cm into the internal carotid artery to reach the bifurcation of middle cerebral artery. Finally, the incision was closed and disinfected. Surgery that only made the cut and artery separation without thread insertion was done in SHAM group rats. Rats were grouped according to random digits table generated by computer software. 4 groups were randomly generated according to the table, including SHAM, pMCAo, pMCAo + BEC-EVs (1 × 10^10^ BEC-EVs), and pMCAo + BMSC-EVs (1 × 10^10^ BMSCs-EVs). Rats with the modified neurological severity score (mNSS) above 6 and Bederson score above 2 post surgery were considered to be successful modeled, and those without successful established pMCAo or dead were excluded in this study. BEC-EVs and BMSC-EVs at the amount of ~ 1 × 10^10^ particles were intravenously administered once via tail vein immediately after surgery whereas PBS without EVs was employed in pMCAo group with the same routine. Total 65 rats were used in this study, and 9 rats were dead before 24 h after surgery, which were excluded from the study. For TTC staining and Evans blue (EB) leakage experiments, 8 rats per groups were applied, and 3 rats per group for brain microvessels isolation, western blotting, and immunofluorescence. 10 rats per group for neurological function evaluation. During the pMCAo surgery, all procedures were conducted gently by skilled investigators, and animal body temperature were kept at 37 ℃ by warm pad to avoid body temperature loss. All rats were kept and treated evenly in an animal room with 12 h of light/dark cycle lighting environment under 20–25 °C room temperature and 50–60% humidity for 24 h. Drug administration order for different rats were randomized in every individual experiment to minimize confounders. Foods and water were freely available in cages after the surgery and EVs treatment, and the rats were euthanized by CO_2_ inhalation at the end of the study.

### Brain cortex microvessels isolation

Rat brain microvessels (BMV) were isolated based on previously reported methods [27], and all procedures were conducted on ice. Briefly, brain samples were collected and shortly preserved in MCDB 131 medium (10372019, Gibco). After white-matter was roughly removed, cortex samples were homogenized and centrifuged (2000 g, 5 min at 4 °C). The supernatant was discarded, and the pellet was resuspended in 15% dextran (MW ~ 70 kDa; 31,390, Sigma-Aldrich). After centrifuged at 10,000 g for 15 min under 4 °C, the pellets with enriched BMV were achieved for further analysis. The isolated BMV was identified by immunofluorescence staining and western blotting.

### The evaluation of neurological function, infarct volume and EB leakage

Neurological function in rats was measured by Bederson score (5-points scoring scale) and mNSS (18-points scoring scale) as described before [28, 29]. Scores in each group were recorded immediately at 6 h, 12 h and 24 h after pMCAo by trained investigators who were blinded to the experiment design. The detailed score scales were provided in (Additional file 1). For brain infarct volume evaluation, 2% 2,3,5-Triphenyl tetrazolium chloride (TTC, T819366, Macklin) were used to stain 2 mm coronal slices under 37 ℃ for 20 min avoiding light. ImageJ (Version 1.53f51, NIH) software was used to calculate the ratio of infarct area to the whole brain area. For EB leakage analysis, rats were intravenously administered 2% EB (4 ml/kg) and allowed to circulate for 1 h. Rats were sacrificed by intraventricularly perfused with 50 ml cold PBS under anesthesia. Whole brains were collected and imaged by IVIS® Spectrum small animal image system (PerkinElmer) at excitation wavelength: 620 nm and emission wavelength: 710 nm.

### BECs permeability assay

5 × 10^4^/well b. End3 cells were seeded into upper chamber with 0.4 μm pore sized 24-well transwell inserts (11820050, Costar) and cultured for 72 h to reach confluence. Before OGD stimulation, DMEM and glucose deprived DMEM (11966–025, Gibco) containing 2 mg/ml TRITC-Dextran (4.4 kDa; T1037, Sigma-Aldrich) were added into upper inserts in Control and OGD groups, respectively, and glucose deprived DMEM medium was added into every lower chamber. Subsequently, EVs from two sources of cells were diluted in glucose deprived culture medium and added to upper chambers in treatment groups, while equal volume of culture medium was added to normalize the chamber volume in other groups. After OGD 4 h, 50 μl medium of each upper and lower chambers was collected, and TRITC-dextran fluorescence intensity was read by microplate reader (SpectraMax M5) at wavelength of excitation: 550 nm and emission: 572 nm. The permeability coefficient was calculated by the following method as previously reported [30]:

$${\text{P}}_{{{\text{dextran}}}} \, = \,\left( {{\text{RFU}}_{{\text{lower chamber}}} /{\text{ RFU}}_{{\text{upper insert}}} } \right) \, \left( {{1 }/{\text{ S}}} \right) \, \left( {\text{V}} \right) \, ({1 }/{\text{ t}}).$$

“RFU” was the fluorescent intensity of upper insert and lower chamber, and “S” indicated the surface area of cell monolayer, while “V” was the volume of lower chamber and “t” represented the time TRITC-dextran spread.

### Western blotting

Total protein extracts of cell samples, BEC-EVs and BMSC-EVs, and isolated brain microvessels were collected, and ProteoExtract Subcellular Proteome Extraction Kit (539790, Calbiochem) was used to extract subcellular protein components in cytosolic fraction (CF), membrane fraction (MF), and actin cytoskeletal fraction (ACF) followed manufacture’s protocol. Samples with equal protein concentration were boiled, electrophoresis separated by 10% sodium dodecyl sulfate (SDS) polyacrylamide gels and transferred into 0.22 μm pore sized polyvinylidene difluoride (PVDF) membranes (1620177, Bio-Rad). After blocking in 5% skimmed milk, membranes were incubated with primary and secondary antibodies, and then washed by TBS-T (Tris-buffered saline with 0.1% Tween 20). ChemiDoc MP Imaging System (Bio-Rad) were applied for imaging. Bands grey value was calculated by ImageJ software and relative expression level of proteins was presented as ratio of β-actin or Fractions REF (Calpain I, Calnexin, Vimentin). Antibodies used in this study were as follows: ZO-1 (1:1000, 61–7300, Invitrogen); Claudin-5 (1:1000, 34–1600, Invitrogen); CD 31 (1:1000, ab281583, Abcam); NeuN (1:5000, ab104225, Abcam); β-actin (1:5000, ab8227,Abcam); Caveolin-1 (1:1000, ab2910, Abcam); TSG 101 (1:1000, ab125011, Abcam); HSP 70 (1:1000, ab137680, Abcam); Calpain I (1:1000, ab108400, Abcam); Calnexin (1: 1000, ab22595, Abcam); Vimentin (1:1000, ab92547, Abcam); Goat Anti-Rabbit IgG H&L (HRP) (1:3000, ab6721, Abcam); Goat Anti-Mouse IgG H&L (HRP) (1:3000, ab67879, Abcam); Normal rabbit IgG (1 μg/ml, 2729S, Cell Signaling Technology).

### Co-immunoprecipitation (Co-IP) assay

Cell samples were lysed by lysis buffer (P0013, Beyotime) with proteinase inhibitor cocktail, and then total protein was extracted, and concentration was evaluated as the protocol of western blotting. Protein G-Magnetic Beads (HY-K0204, MCE) were conjugated with Anti-Cav-1 antibodies or Anti-Normal rabbit IgG for 2 h under 4 ℃. After magnetic separation and washed by PBS-T (0.5% Tween-20 in PBS, pH 7.4) for 4 times, and 300 μg cell total proteins were added to beads and incubated overnight at 4 ℃. Excessive antigens were washed by PBS-T, and conjugated antigens were eluted by heating under 95 ℃ for 5 min with loading buffer and separated by electrophoresis as western blotting.

### Immunofluorescence staining

For immunofluorescence samples preparation, rats were sacrificed, and then, 50 ml cold PBS and 50 ml cold 4% paraformaldehyde (PFA) were intraventricularly perfused slowly. Whole brains were collected, fixed by 4% PFA and dehydrated in 15% and 30% sucrose solution. 10 μm brain cryosections were cut by microtome (CryoStar NX70, Thermo Fisher Scientific). Brain microvessels were resuspended in PBS and dropped at glass slides after isolation, and samples were prepared after air dry. Cell samples were prepared in confocal dish after treatment. All samples were washed by PBS, fixed by 4% PFA and permeabilized by 0.1% Triton X-100. Primary and secondary antibodies (Alexa Fluor® 488 or 594 of rabbit or mouse, 1:500, ab150113; ab150080; ab150077, Abcam) were incubated with samples, and then DAPI was stained for nucleus before sections were sealed to be imaged by confocal (Lecia, SP8) or fluorescent microscope (Lecia, DMi8) under 63 × , 10 × , and 40 × objectives for cells, brain slices and microvessels, respectively. Images were processed by the microscopy software LAS X (Lecia), and fluorescence intensity was measured by ImageJ software. The following primary antibodies were used: ZO-1 (1:200, 61–7300, Invitrogen); Claudin-5 (1:150, 34–1600, Invitrogen), Caveolin-1 (1:250, ab2910, Abcam); CD31 (1:200, ab64543, Abcam).

### Cav-1 siRNA and pcDNA 3.1 vector transfection

Cav-1 siRNA/pcDNA 3.1 and FAM-negative control siRNA/pcDNA 3.1-GFP (GenePhrama) were transfected by utilizing lipofectamine 3000 reagent (L3000015, Invitrogen) according to the manufacturer’s protocol. Cav-1 knockdown and overexpression were verified by western blotting and GFP/FAM labels imaging under 10 × objective by fluorescent microscope (Lecia, DMi8). Detailed pcDNA 3.1 vector gene map has been provided in (Additional file 1).siRNA sequences applied:

Cav-1 (sense 5'-3'): CUGCGAUCCACUCUUUGAATT.

Cav-1 (antisense 5'-3'): UUCAAAGAGUGGAUCGCAGTT.

Negative Control siRNA oligo (sense 5'-3'): UUCUCCGAACGUGUCACGUTT.

Negative Control siRNA oligo (antisense 5'-3'): ACGUGACACGUUCGGAGAATT.

Cav-1 gene information applied in pcDNA 3.1 vector: NCBI Gene ID:12389.

### Transmission electron microscopy

TEM was employed to observe the morphology of the EVs and the rat brain microvessel microstructure. Briefly, for EVs samples, 20 μl of EVs suspension was dropped onto the 150 meshes carbon filmed copper grid for 5 min. Thereafter, 2% phosphotungstic acid was dropped on the copper grid to stain for 2 min. The samples were observed under TEM (HT7800, Hitachi) at the 40 k × magnification. For rat microvessel, 1 mm^3^ rat cortex brain tissues were harvested and fixed in 2.5% glutaraldehyde solution and 1% OsO_4_. Afterward, samples were dehydrated by using 30%-95% ethanol, and then subjected to resin penetration and embedding. The embedded samples were cut into 60 nm section by using the ultra-microtome (Leica UC7, Leica). Tissue samples were fished out onto the 150 meshes formvar filmed cuprum grids and stained by 2% uranium acetate saturated alcohol solution for 8 min. After rinsed in 70% ethanol, and ultra-pure water for 3 times, respectively, samples were then stained by 2.6% lead citrate for 8 min, followed by rinsed in ultra-pure water 3 times. Sections were dried overnight and observed under TEM with the magnification of 2 k × or 20 k × . ImageJ software was used to quantify the caveolae structure density.

### Statistical analysis

All data of this study were collected and analyzed by GraphPad Prism 8 software and presented as mean ± standard deviation (SD). Statistical significance was considered when p value < 0.05. Additionally, two-way ANOVA was performed in experiments which involve two variations. All data were normalized and calculated as fold of Control group.

## Results

### Characteristics of BMSC-EVs and BEC-EVs

**Fig. 1:**
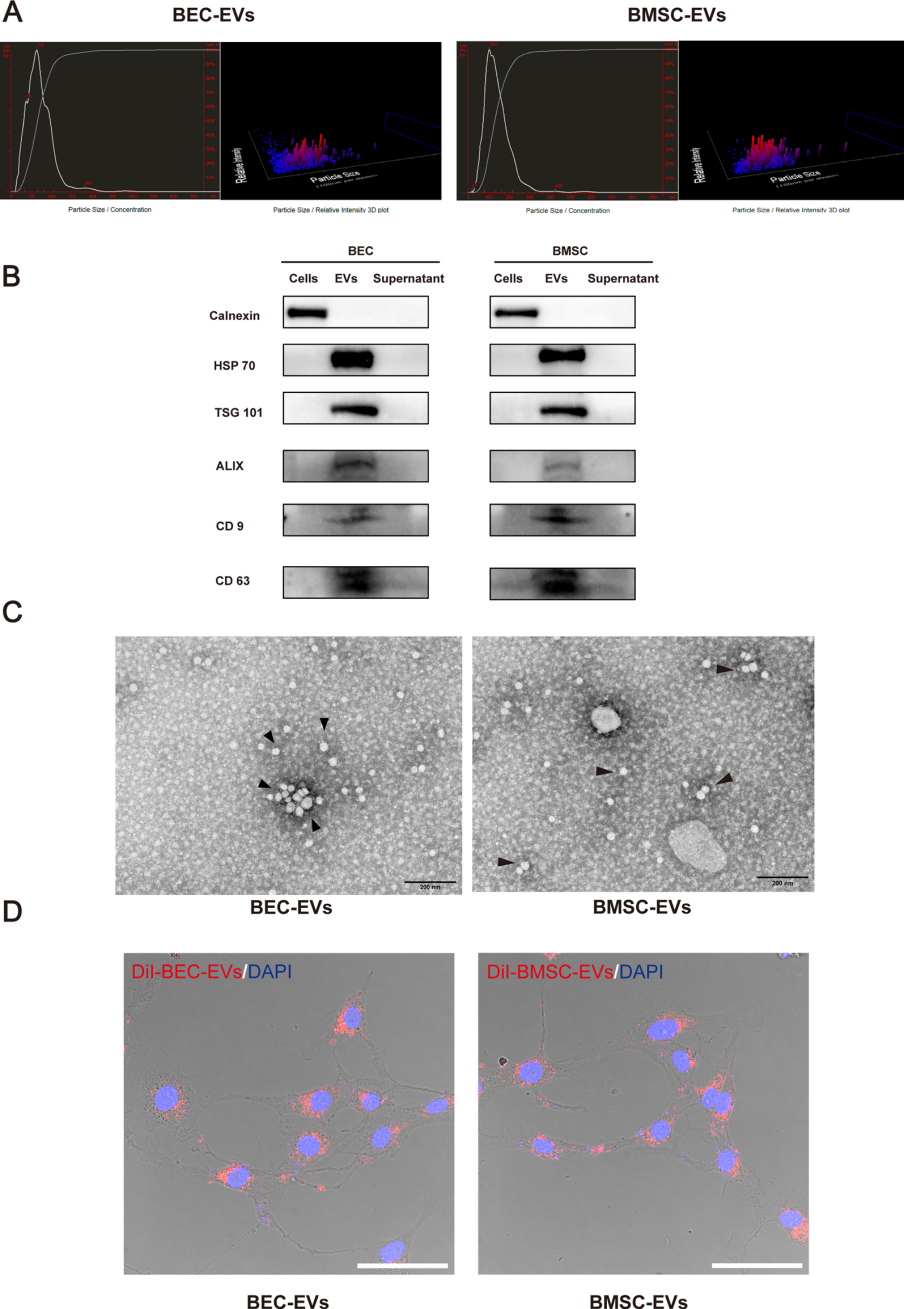
Characterization of BEC-EVs and BMSC-EVs. **A** NTA of BEC-EVs and BMSC-EVs showed the size of EVs. **B** Representative western blotting of EVs marker HSP 70, TSG 101, ALIX, CD9, and CD63. Cells and supernatant without EVs were regarded as negative control, respectively, and calnexin was defined as endoplasmic reticulum marker which presented exclusively in cells. **C** Representative TEM images of BEC-EVs and BMSC-EVs (Black arrowheads). Scale Bar: 200 nm. Magnification: 40 k × . All images were taken in the same scale. **D** Representative confocal microscope images of b. End3 cells phagocytizing DiI labeled BEC-EVs and BMSC-EVs. Scale Bar: 50 μm. Magnification: 63 × . All images were taken in the same scale

To identify the isolated EVs, the size distribution, morphology, and the phagocytized ability were characterized. The average sizes of BMSC-EVs and BEC-EVs were 145 nm and 142 nm as determined by NTA (Fig. 1A), whereas negative control sample showed no typical EVs distribution, and had extremely low nanoparticle tracks (less than 200 tracks) (Additional file 2: Fig. S1A) and EVs surface markers TSG 101, HSP 70, Alix, CD 9 and CD 63 were exclusively expressed in EVs from two sources of cells whereas were not detected in parent cells and supernatant after EVs isolation (Fig. 1B). TEM images indicated EVs had an intact sphere structure (Fig. 1C). In contrast, negative control samples had no obvious nanoparticle feedback (Additional file 2: Fig. S1B). Notably, DiI labeled BMSC-EVs and BEC-EVs could be phagocytized by b. End3 cells (Fig. 1D), and DiI labeled negative control nearly failed to be observed in b. End3 cells, which indicated no residue of excessive DiI dye (Additional file 2: Fig. S1C). Additionally, DiI labeled BMSC-EVs and BEC-EVs were internalized by b. End3 cell over time under 37 ℃, however, the cells failed to phagocytize DiI labeled EVs at any time point within 120 min under 4 ℃, which indicated the EVs intracellular internalization was affected by non-physiological ambient temperature (Additional file 2: Fig. S1D). Collectively, the results indicated the isolated EVs preserved ideal size, morphology, and the ability of intracellular internalization.

### BMSC-EVs and BEC-EVs treatments attenuated BECs hyperpermeability after OGD insult

**Fig. 2:**
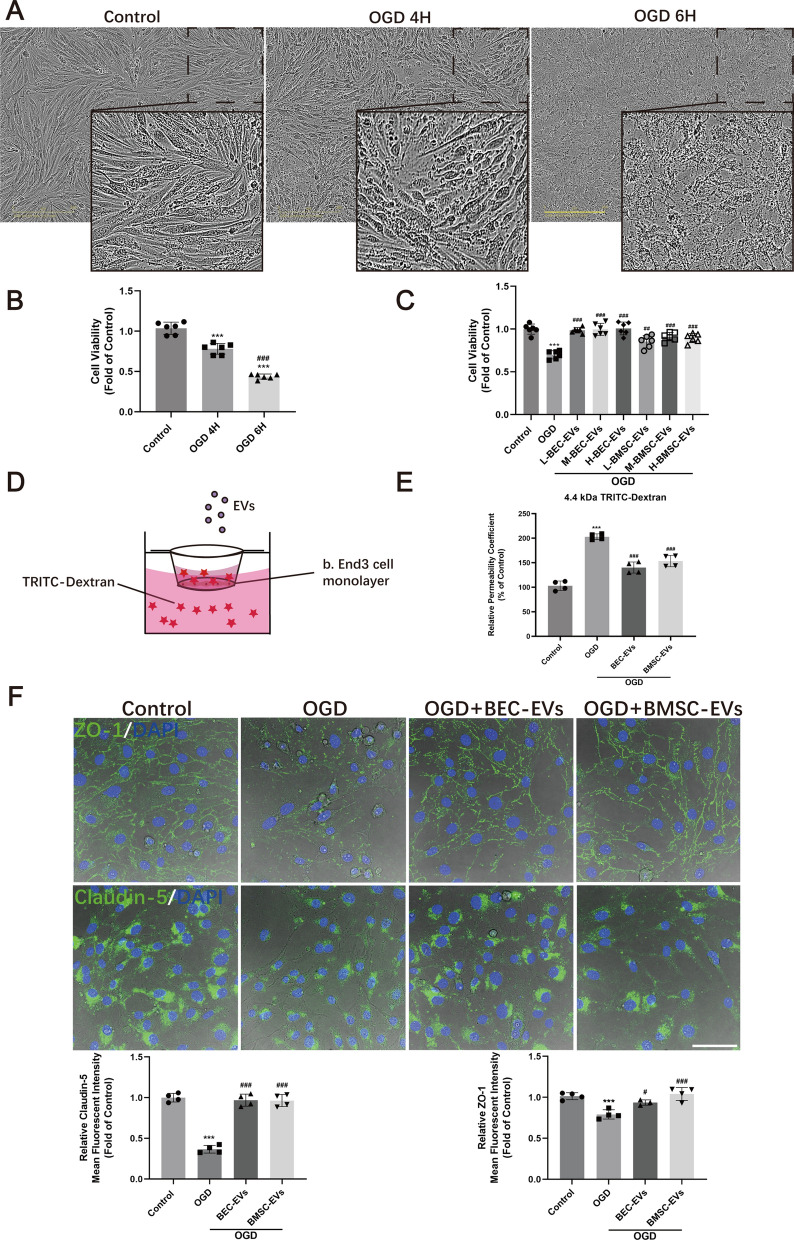
BEC-EVs and BMSC-EVs treatments reduced the leakage and enhanced the expressions of ZO-1 and Claudin-5 in OGD insulted b. End3 cells. **A** Representative images of b. End3 cell morphology after 4 and 6 h OGD insult. Scale Bar: 200 μm. Magnification: 20 × . All images were taken in the same scale. **B** The viability analysis of OGD insulted b. End3 cells after BEC-EVs and BMSC-EVs treatments (n = 6). **C** Viability analysis of b. End3 subjected to OGD after different dosages of BEC-EVs and BMSC-EVs administrations (n = 6). **D** Schematic of transwell insert for the evaluation of TRITC-Dextran leakage after the treatments of EVs from two sources. **E** Relative permeability coefficient of OGD insulted b. End3 cells after BEC-EVs and BMSC-EVs treatments (n = 4). **F** Representative immunofluorescent staining images of ZO-1 and Claudin-5 in OGD insulted b. End3 cells after BEC-EVs and BMSC-EVs treatments, and quantification of mean fluorescent intensity. Scale Bar: 50 μm. Magnification: 63 × . All images were taken in the same scale. ^***^P < 0.001 vs. Control group; ^###^*P* < 0.001, ^##^*P* < 0.01, ^#^*P* < 0.05 vs. OGD or OGD 4 h group

To evaluate the in vitro efficacy of BBB integrity exerted by two kinds of EVs, OGD stimulated b. End3 cells were treated with BMSC-EVs and BEC-EVs. The morphology of b. End3 cells gradually presented injury with soma shrinkage and rupture, after OGD stimulation for 4 or 6 h (Fig. 2A). Consistent with the alteration of cell morphology, cell viability was decreased by about 20% to 60% after 4 to 6 h OGD stimulation (Fig. 2B). As cells morphology was extremely upset under 6 h OGD insult, we chose 4 h OGD for the following studies. Then, to determine the optimal dosages of EVs from two sources of cells, low, medium, and high doses of BEC-EVs and BMSC-EVs were applied to evaluate cell viability. The results suggested three dosages of BEC-EVs presented cell viability reservation by about 30% whereas three dosages of BMSC-EVs recovered cell viability by about 20% against hypoxic insult (Fig. 2C), and no significant difference existed between three doses of BMSC-EVs and BEC-EVs groups (low dosage: ~ 5 × 10^9^ EVs; middle dosage: ~ 1 × 10^10^ BEC-EVs; high dosage: ~ 2 × 10^10^ EVs). Medium dosage of EVs was determined for the following experiments to achieve stable therapeutic outcomes. In addition, the leakage of monolayer was dramatically increased after OGD insult by transwell assay (Fig. 2D, E), and the hyperpermeability of OGD insulted cell monolayer was attenuated about 30% by BMSC-EVs and BEC-EVs administrations (Fig. 2E).

### BMSC-EVs and BEC-EVs treatments enhanced ZO-1 and Claudin-5 expressions and inhibited their redistribution in OGD insulted BECs

**Fig. 3:**
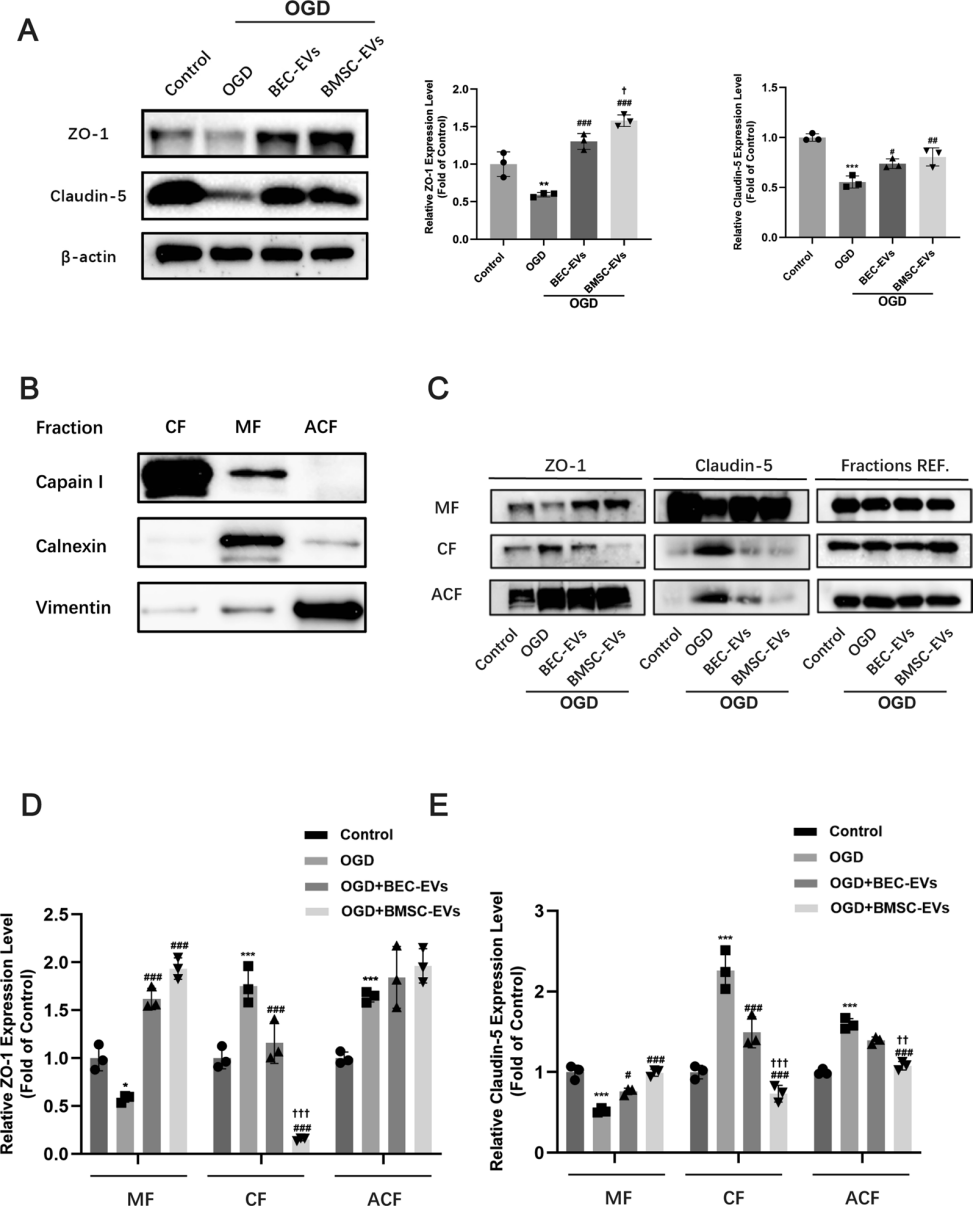
BEC-EVs and BMSC-EVs treatments recovered ZO-1 and Claudin-5 expressions and inhibited their intracellular translocation in OGD insulted b. End3 cells. **A** Representative western blotting of ZO-1 and Claudin-5 after the treatments of EVs from two sources and quantification of ZO-1 and Claudin-5 expressions (n = 3). **B** The purity determination in subcellular fractions by western blotting. **C** Representative western blotting of ZO-1 and Claudin-5 in subcellular fractions. *MF* Membrane fraction; *CF* Cytoplasm fraction; *ACF* Actin cytoskeleton fraction; Fractions REF. (Calpain I for AF, Calnexin for MF and Vimentin for ACF, respectively). **D**, **E** Quantification of ZO-1 and Claudin-5 expressions in subcellular fractions (n = 3). ^***^P < 0.001, ^**^P < 0.01, ^*^P < 0.05 vs. Control group; ^###^*P* < 0.001, ^##^*P* < 0.01, ^#^*P* < 0.05 vs. OGD group; ^†††^*P* < 0.001, ^††^*P* < 0.01, ^†^*P* < 0.05 vs. OGD + BEC-EVs group

We next assessed the EVs’ therapeutic effects on the expression and dislocation of tight junction proteins in b. End3 cells after OGD insult. Immunofluorescence staining of ZO-1 and Claudin-5 showed that BMSC-EVs and BEC-EVs treatments had similar efficacies to increase their fluorescence intensity in OGD insulted b. End3 cells (Fig. 2F). Western blotting results also suggested the similar trend by the treatments of EVs from two sources, especially by BMSC-EVs treatment (Fig. 3A). Additionally, subcellular membrane, cytoplasm, and actin cytoskeleton fractions proteins from b. End3 cells were isolated, and the subcellular fraction markers Calpain I, Calnexin, and Vimentin were dominantly expressed in corresponded fractions (Fig. 3B), suggesting the extraction purity was reliable. The expressions of ZO-1 and Claudin-5 in membrane fraction were decreased after OGD insult, which were significantly reversed by BMSC-EVs and BEC-EVs treatments. Simultaneously, the expressions of ZO-1 and Claudin-5 were dramatically increased in cytoplasm whereas the treatments of EVs from two sources, especially BMSC-EVs treatment reversed this trend (Fig. 3C–E). In actin cytoskeleton, only Claudin-5 expression were downregulated by BMSC-EVs treatment (Fig. 3E). Altogether, the results suggested BMSC-EVs treatment more efficiently antagonized the redistribution of ZO-1 and Claudin-5 from cellular membrane to cytoplasm and actin cytoskeleton after OGD stimulation.

### BMSC-EVs and BEC-EVs treatments increased ZO-1 and Claudin-5 expressions in isolated cerebral microvessels

**Fig. 4:**
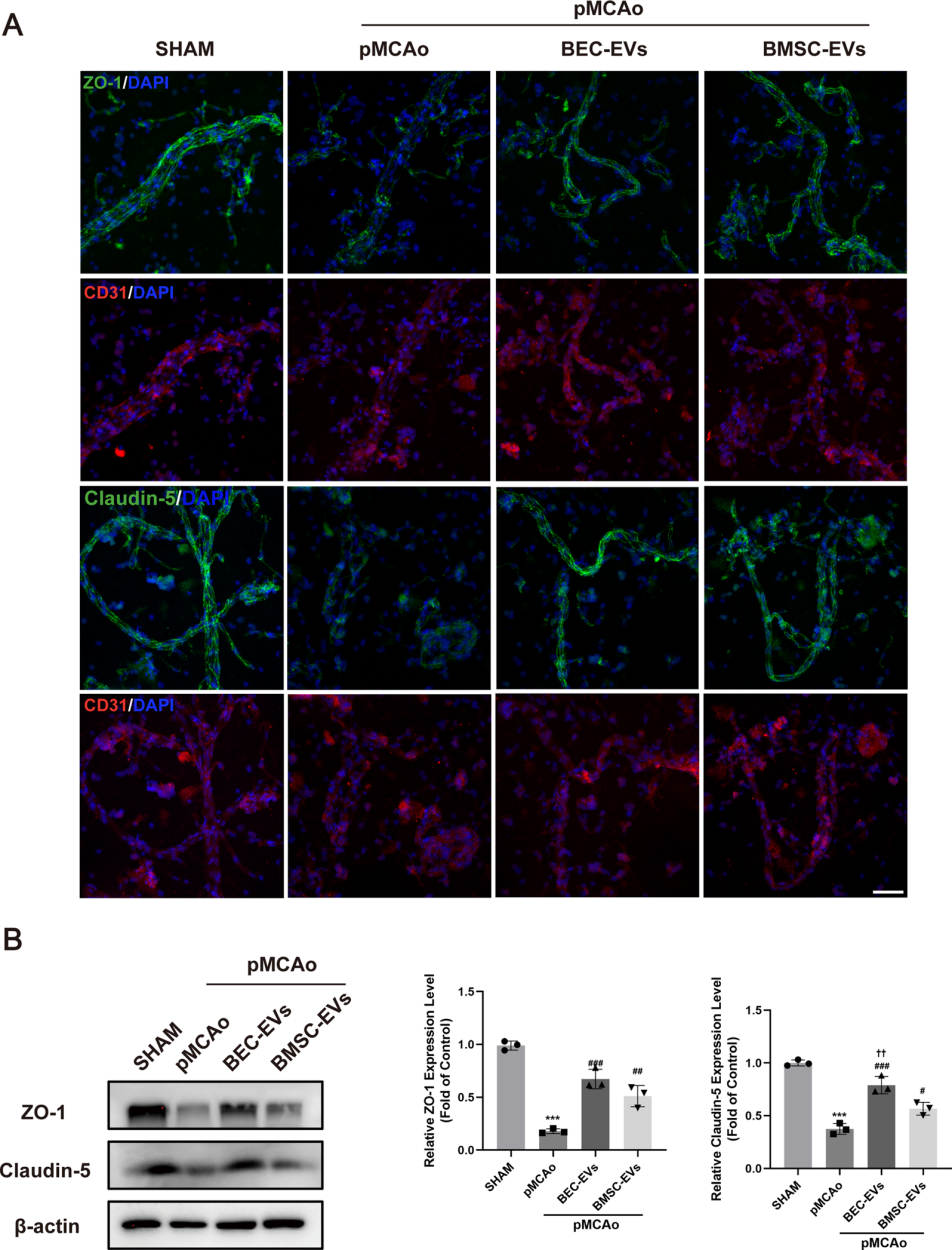
BEC-EVs and BMSC-EVs treatments enhanced ZO-1 and Claudin-5 expressions in BMV from ischemic cortex. **A** Representative immunofluorescent staining of Claudin-5, ZO-1 and CD31 in BMV. Scale Bar: 50 μm. Magnification: 40 × . All images were taken in the same scale. **B** Representative western blotting of Claudin-5 and ZO-1 in BMV and quantification of ZO-1 and Claudin-5 expressions (n = 3). ^***^P < 0.001 vs. SHAM group; ^###^*P* < 0.001, ^##^*P* < 0.01, ^#^*P* < 0.05 vs. pMCAo group; ^††^*P* < 0.01 vs. pMCAo + BMSC-EVs group

Furthermore, brain microvessels (BMV) from rat ischemic cortex were isolated. BMV from normal cortex was identified by western blotting and immunofluorescence staining, as the results presented, NeuN (a neuron marker) was highly expressed in brain total extract (BTE), while it was almost absent in BMV (Additional file 3: Fig. S2A). Moreover, the vascular marker CD 31 mainly expressed in BMV by western blotting and immunofluorescence staining, suggesting the successful microvessels isolation (Additional file 3: Fig. S2A-B). After 24 h pMCAo, the fluorescence intensity of Claudin-5 and ZO-1 presented weak and unclear in BMV from ischemic cortex post-stroke, while partly salvaged by BMSC-EVs and BEC-EVs treatments (Fig. 4A). To quantify such differences, western blotting of BMV was performed, and Claudin-5 and ZO-1 expressions were extremely lowered after pMCAo, however, the treatments of EVs from two sources of cells reversed this trend. Moreover, the expression of total protein of Claudin-5 in BMV in BEC-EVs group was more significant when compared with that in BMSC-EVs group (Fig. 4B).

### BMSC-EVs and BEC-EVs treatments rescued pMCAo rats against ischemic injury and BBB leakage

**Fig. 5:**
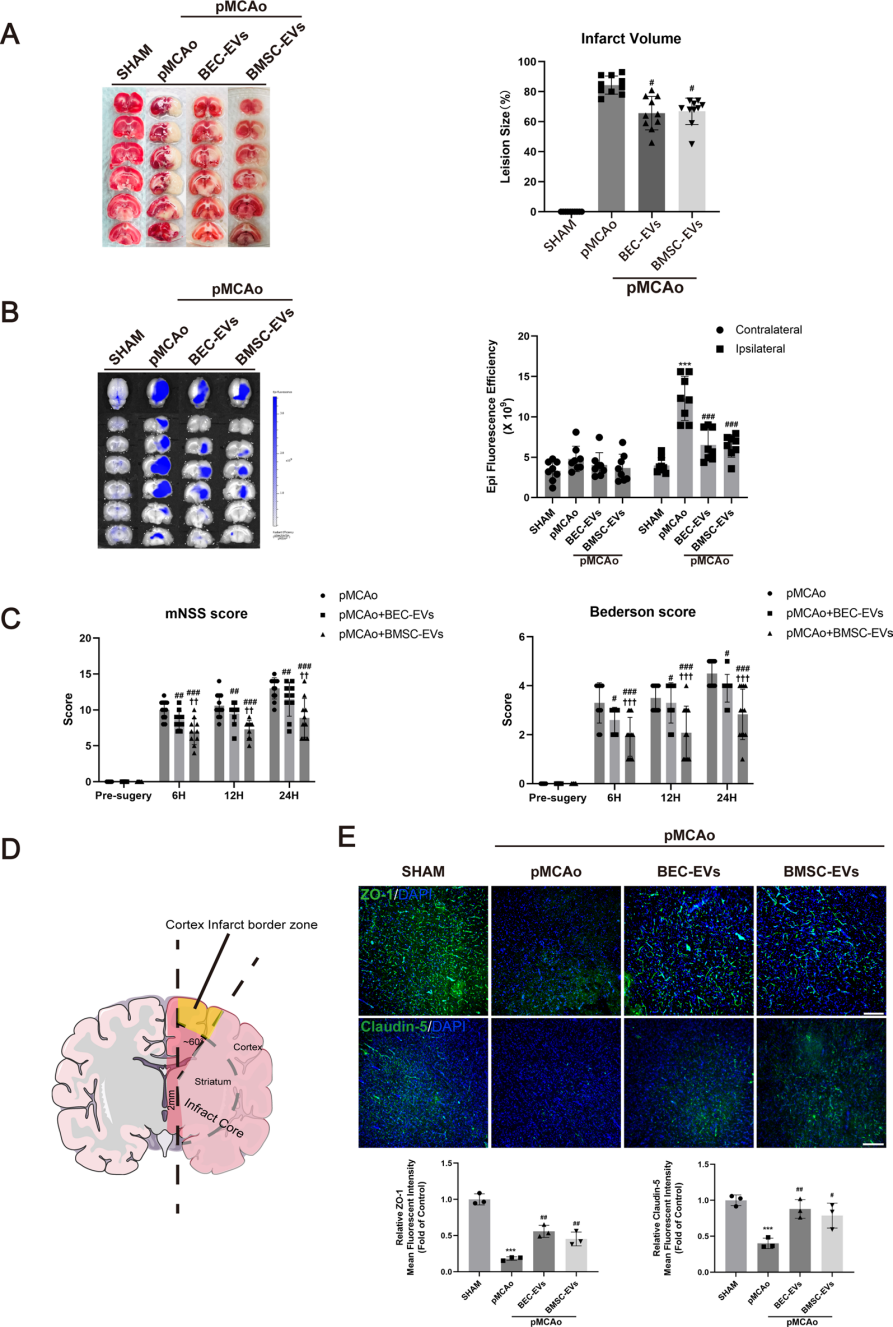
BEC-EVs and BMSC-EVs treatments improved neurological injury, BBB leakage and ZO-1 and Claudin-5 expressions after acute IS. **A** Representative TTC staining images of total brain slices from pMCAo rats and relative infarction volume quantification (n = 8). **B** Representative images of EB leakage determined by IVIS and quantification of fluorescent efficacy (n = 8). **C** The evaluation of neurological function by mNSS and Bederson tests (n = 10). **D** Schematic of cortex infarct border area observed in immunofluorescent staining. **E** Representative immunofluorescent staining and expression quantification of ZO-1 and Claudin-5 in cortex infarct border area (n = 3). Scale Bar: 200 μm. Magnification: 10 × . All images were taken in the same scale. ^***^P < 0.001 vs. SHAM group; ^###^*P* < 0.001, ^##^*P* < 0.01, ^#^*P* < 0.05 vs. pMCAo group; ^†††^*P* < 0.001, ^††^*P* < 0.01, vs. pMCAo + BEC-EVs

Next, we further evaluated the therapeutic efficacy of EVs in vivo, and administered two kinds of EVs to pMCAo rats. After the administrations of BMSC-EVs and BEC-EVs for 24 h, respectively, cerebral infarct volume, neurological function and BBB permeability were evaluated. It displayed similar effects on the reduction of infarct volume and EB leakage in ischemic ipsilateral hemisphere (Fig. 5A, B). Deficient neurological function evaluated by mNSS and Bederson tests was partly ameliorated by the administrations of EVs from two sources, and particularly, the efficacy of BMSC-EVs treatment was superior to that of BEC-EVs treatment (Fig. 5C). In addition, the fluorescence intensity of ZO-1 and Claudin-5 in infarct border zone was observed according to previous report [31]. Briefly, we made a vertical cut at about 2 mm from the midline in ischemic hemisphere, and then made a cut at 60° from midline to separate ischemic core from infarct border zone (Fig. 5D). The intensity of ZO-1 and Claudin-5 was dramatically decreased after pMCAo, which was restored by the treatments of EVs from two sources (Fig. 5E), and the improved trend was similar to that in BMV (Fig. 4A).

### BMSC-EVs and BEC-EVs treatments antagonized Cav-1-dependent ZO-1 and Claudin-5 endocytosis

**Fig. 6:**
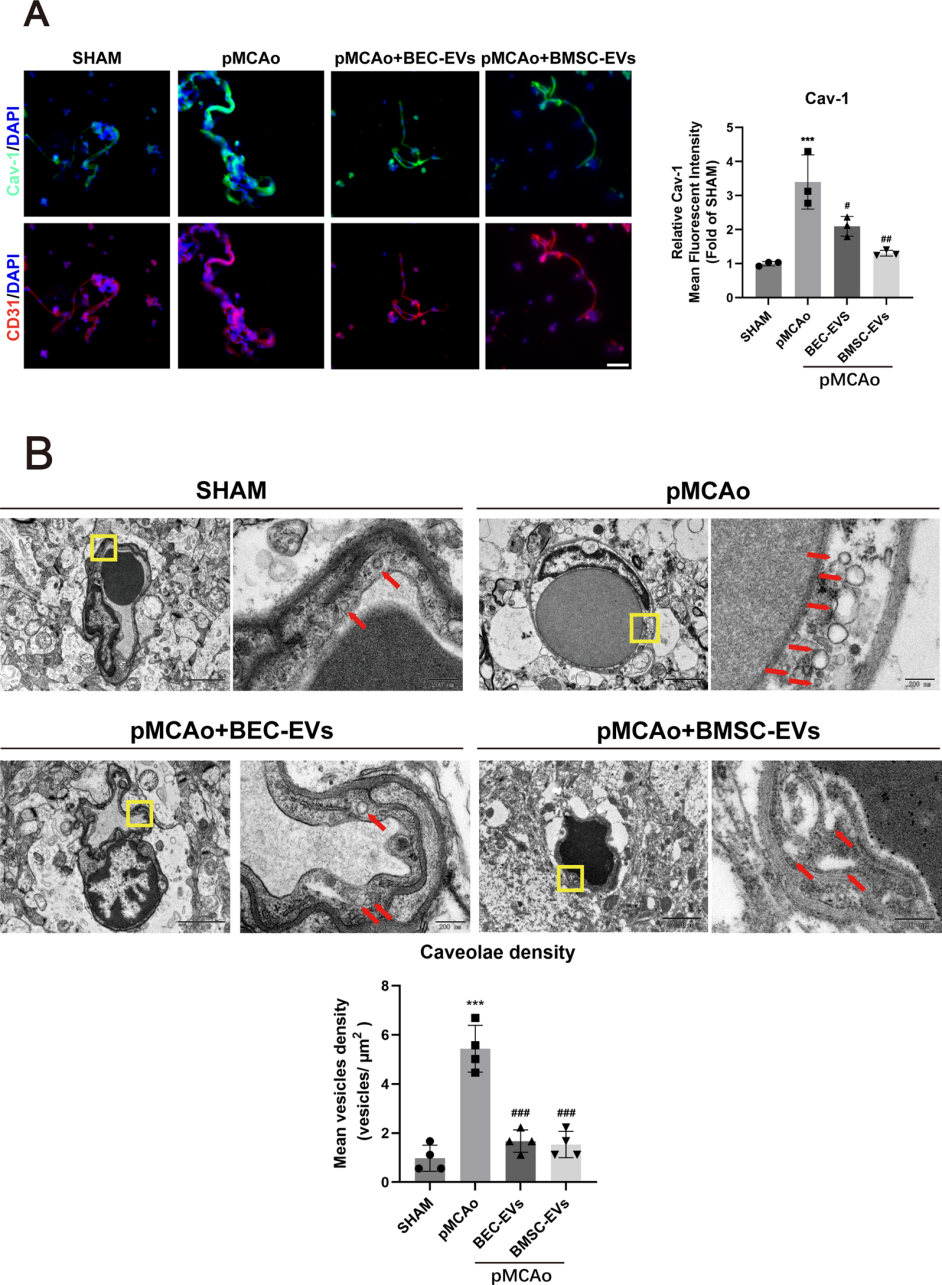
Distribution and expression of Caveolin-1 and Caveolae in brain microvessels. **A** Representative immunofluorescence staining images and quantification of Caveolin-1 and CD 31 in BMV (n = 3). Scale Bar: 50 μm. Magnification: 40 × . All images were taken in the same scale. **B** TEM image of caveolae-like vesicles in brain microvascular endothelial cells and quantification of the density. Scale Bar: 2 μm and 200 nm. Magnification: 2 k × and 20 k × . All images were taken in the same scale. ^***^P < 0.001 vs. SHAM group; ^###^*P* < 0.001, ^##^*P* < 0.05, ^#^*P* < 0.01 vs. pMCAo group

**Fig. 7:**
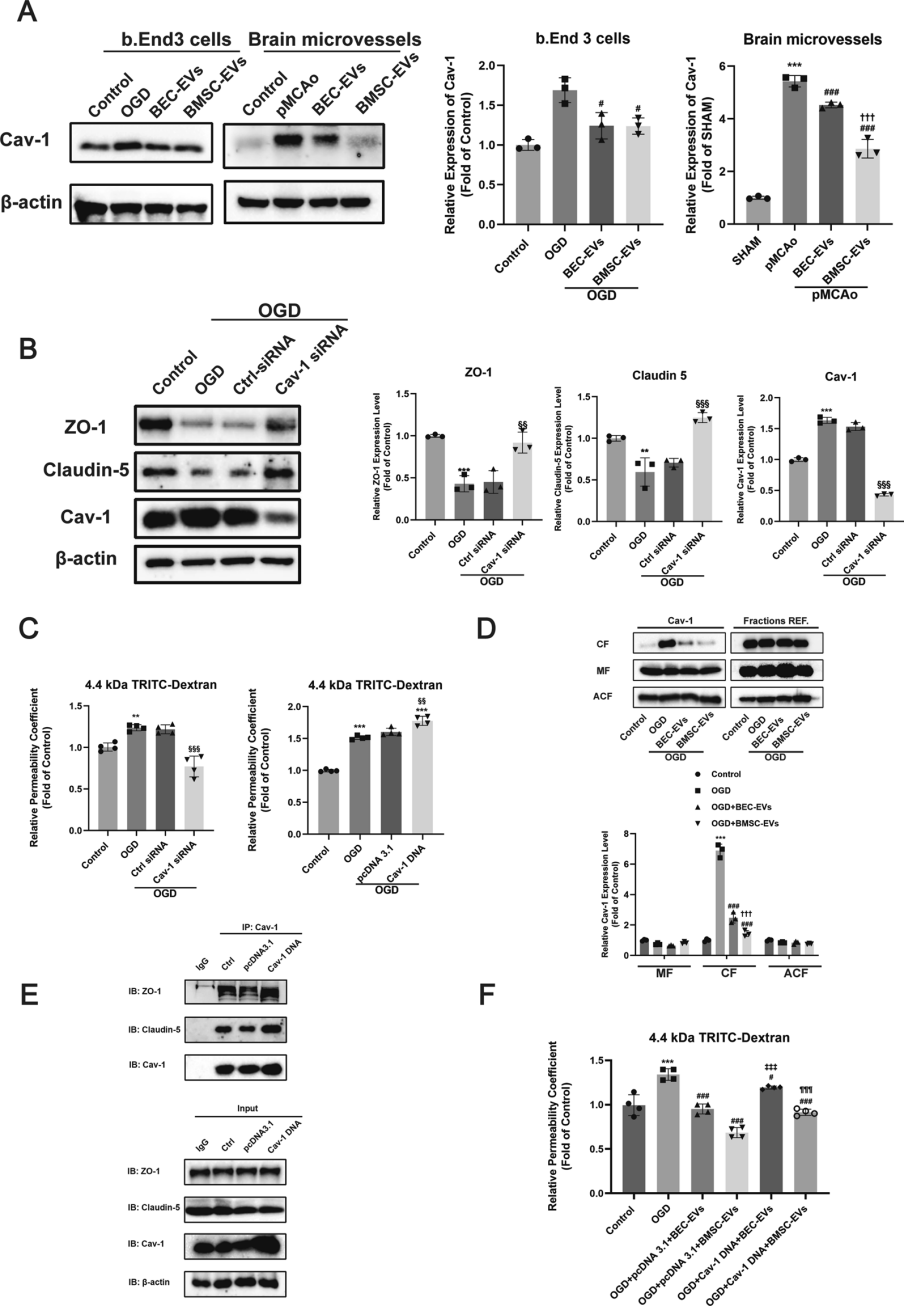
BEC-EVs and BMSC-EVs treatments antagonized Cav-1-dependent ZO-1 and Claudin-5 endocytosis to decrease BBB permeability. **A** Representative western blotting of Cav-1 in OGD insulted b. End3 cells and BMV from ischemic cortex; and quantification of Cav-1 expression (n = 3). **B** Representative western blotting of ZO-1, Claudin-5 and Cav-1 after Cav-1 siRNA transfection in OGD insulted b. End3 cells and quantification of ZO-1, Claudin-5 and Cav-1 expressions (n = 3). **C** Relative permeability coefficient of OGD insulted b. End3 cells after siRNA/pcDNA 3.1 transfection (n = 4). **D** Representative western blotting of Cav-1 in subcellular fractions. *MF* Membrane fraction; *CF* Cytoplasm fraction; *ACF* Actin cytoskeleton fraction; Fractions REF. (Calpain I for AF, Calnexin for MF and Vimentin for ACF, respectively) and quantification of Cav-1 expression in subcellular fractions (n = 3). **E** Representative co-ip results of Cav-1 with ZO-1 and Claudin-5 after Cav-1 pcDNA 3.1 transfection in b. End3 cells, and IgG was served as negative control. IP: Immunoprecipitation; IB: Immunoblotting. **F** Relative permeability coefficient of OGD insulted b. End3 cells with pcDNA 3.1 transfection after the treatments of EVs from two sources (n = 4). ^***^P < 0.001, ^**^P < 0.01 vs. Control/SHAM group; ^###^*P* < 0.001, ^#^*P* < 0.05 vs. pMCAo/OGD group; ^†††^*P* < 0.0001, vs. pMCAo + BEC-EVs. ^§§§^*P* < 0.001, ^§§^*P* < 0.01 vs. Ctrl-siRNA/pcDNA 3.1 group. ^‡‡‡^*P* < 0.001 vs. OGD + pcDNA 3.1 + BEC-EVs group; ^¶¶¶^*P* < 0.001 vs. OGD + pcDNA 3.1 + BMSC-EVs group

To investigate the pros and cons of Cav-1 on BBB permeability, we focused on the relationship between Cav-1 and TJ proteins. Results showed the expression of Cav-1 in OGD insulted b. End3 cells and BMV from ischemic cortex was obviously upregulated, while this trend was reversed by BEC-EVs and BMSC-EVs treatments (Fig. 6A), and TEM image revealed that the density of caveolae-like vesicles in pMCAo rats’ brain microvascular endothelial cells were notably accumulated but dwindled by two kinds of EVs therapies (Fig. 6B). Consistent with immunofluorescence staining result in BMV, western blotting of the increased Cav-1 in BMV of pMCAo model was downregulated in EVs administration groups (Fig. 7A). Interestingly, the decreased expressions of ZO-1 and Claudin-5 in OGD insulted b. End3 cells were recovered by Cav-1 siRNA transfection (Fig. 7B). Additionally, it also indicated Cav-1 knockdown, and overexpression were successfully performed in b. End3 cells (Additional file 4: Fig. S3A-B), and attenuating Cav-1 expression by siRNA contributed to the decrease of BEC monolayer permeability, whereas Cav-1 overexpression by Cav-1 pcDNA 3.1 increased the monolayer leakage (Fig. 7C). Furthermore, Cav-1 expression was abundant in MF and ACF, and particularly upregulated in CF after OGD insult, but in a significant manner, it was downregulated by the treatments of EVs from two cell sources, especially by BMSC-EVs treatment (Fig. 7D). Co-IP experiment revealed that the colocalization of Cav-1 with ZO-1 and Claudin-5 was enhanced in b. End3 cells by Cav-1 overexpression (Fig. 7E), and the results showed that Cav-1 interacted closely with ZO-1 and Claudin-5. Finally, to prove the relationship of Cav-1 with EVs therapeutic efficacy, we administered EVs from two sources into Cav-1-overexpressed b. End3 cells insulted by OGD, and as expected, the decreased cell monolayer leakage by BMSC-EVs and BEC-EVs treatments was partly muted in Cav-1 overexpression groups (Fig. 7F). In summary, the results indicated Cav-1 mediated ZO-1 and Claudin-5 endocytosis after OGD insult, which contributed to endothelial hyperpermeability.

## Discussion

In this study, our results demonstrated the adverse role of Cav-1 in BBB permeability in acute IS, which was associated with Cav-1-dependent TJ proteins endocytosis from endothelial cell membrane to cytoplasm. Both BMSC-EVs and BEC-EVs treatments decreased BBB leakage and infarction volume, as well as improved neurological function. By comprehensive comparative analysis, the efficacies of BMSC-EVs treatment on neurological functional amelioration and antagonizing Cav-1-denpendent ZO-1 and Claudin-5 endocytosis are superior to BEC-EVs treatment.

Recently, increasing attentions were attracted on the therapeutic paracrine factors of stem cells rather than themselves [32, 33]. EVs can be released by nearly all kind of cells, and based on Minimal Information for Studies of Extracellular Vesicles 2018 (MISEV2018) [34], our isolated EVs can be considered as small EVs (sEVs) which has a smaller size less than 200 nm. As one of the recognized cells paracrine factors, with protein, RNA and DNA cargos packed by their parent cells, EVs were reported to mediate multiple biological functions and penetrate BBB to improve neurodegeneration/cardiovascular recovery [35]. Therefore, EVs have ridden the new wave of cell-free therapeutic with similar efficacy and tend to be safer than their parent cells [32, 36]. The treatment of EVs derived from BMSCs has been extensively reported to achieve good effects on IS via multiple pathways [24, 37–40], and the treatment of EVs derived from BECs also exhibited satisfying efficacy on experimental IS [22, 41, 42]. However, few studies reported the discrepancy of therapeutic efficacies on BBB integrity between the two cells sources' EVs post-stroke.

In our study, we compared the therapeutic efficacies of BMSC-EVs and BEC-EVs on acute IS in vitro and in vivo, and found that both kinds of EVs had similar abilities to attenuate hyperpermeability of b. End3 cell insulted by OGD, followed by restoring ZO-1 and Claudin-5 expressions and antagonizing their endocytosis abnormality. Moreover, BMSC-EVs treatment more definitely intervened with the redistribution of ZO-1 and Claudin-5 in subcellular fractions than BEC-EVs treatment did. In pMCAo rats, the efficacies of EVs from two sources on BBB leakage were the same as those in vitro. Notably, the therapeutic effect of BMSC-EVs on neurological function was superior to that of BEC-EVs. The potential reason may be attributed to the pluripotent characteristic of BMSCs, which enabled them to produce EVs to exert broad protective functions on diverse NVU cells as previously concluded [40], whereas BEC-EVs derived from BECs which compose an important structure of BBB, may specifically present vascular protective effects. It can be employed to explain why the expressions of ZO-1 and Claudin-5 in BEC-EVs group were higher than those in BMSC-EVs group in BMV.

Cav-1 is a critical scaffolding/regulatory protein of Caveolae in lipid raft and mediates pathophysiological processes including Cav-1-dependent endocytosis [43]. However, its role in regulating of BBB permeability after IS always under controversy in recent years [11, 14]. In rat cortical cold injury model and cerebral I/R model, Cav-1 upregulation occurred before TJs breakdown, and in early stage of ischemia, BBB leakage resulted mainly from Cav-1-dependent transcytosis [10, 44], suggesting Cav-1 is a potential therapeutic target for BBB integrity. Additionally, Cav-1 was also reported to alter the subcellular distribution of TJ proteins. Liu and colleagues found that Cav-1 mediated the redistribution of Claudin-5, which contributed to BBB permeability in early IS phase [13]. Furthermore, their results demonstrated that Claudin-5 was finally degraded by autophagy via the interaction of NO with Cav-1 in OGD-insulted ECs [45]. Similarly, Cav-1, MMP-2/9 and autophagy-lysosome involved ZO-1 intracellular translocation and degradation resulted in BBB hyperpermeability in rats with I/R [46]. Although above results suggested Cav-1 plays an important role in ZO-1 and Claudin-5 redistribution and degradation post-stroke, whether Cav-1 directly or indirectly affects ZO-1 and Claudin-5 is still ambiguous, especially in permanent stroke model.

Currently, our results demonstrated Cav-1 expression was significantly upregulated in OGD-insulted b. End3 cells and BMV from ischemic cortex, and it accumulated in cellular cytoplasm fraction after OGD stimulation. Moreover, by Cav-1 knockdown/overexpression and Co-IP assays, we defined a Cav-1-dependent endocytic pathway for ZO-1 and Claudin-5. Combing with the significant inhibition of Cav-1 in BMV from ischemic cortex in BMSC-EVs group, it suggested that BMSC-EVs treatment had more powerful ability to antagonize Cav-1-dependent ZO-1 and Claudin-5 endocytosis compared with BEC-EVs treatment. However, our results showed that there were no statistical significances in the attenuation of BBB leakage in vivo between BMSC-EVs and BEC-EVs treatments. We hypothesize BEC-EVs treatment regulated other Cav-1 independent approaches to supplement TJ proteins on cellular membrane (e.g., the suppression of MMP2/9, NO generation inhibition and endothelial protection and so on). In present study, we failed to further address “the fate” of the endocytosed ZO-1 and Claudin-5, and based on previous report, autophagy can be the following pathway that is responsible for the degradation of TJ proteins [45, 46]. In future works, the mechanism of how EVs adjust autophagy-lysosome dependent ZO-1 and Claudin-5 degradation should be further explored. Additionally, we also failed to explore what components of EVs could intervene with Cav-1 mediated ZO-1 and Claudin-5 translocation or disruption, and we hypothesize that the potential effects were related to microRNAs (miRNAs) loaded in EVs. miRNAs are small stem-ring structured no-coding RNA, which can be packed in EVs by their parent cells to mediate cell-to-cell communication and multiple biological functions [47]. Therefore, studies had verified some exosomal miRNAs were the key to IS recovery such as miR-126 in endothelial-EVs and miR-17–92 in BMSC-EVs [23]. Further studies should apply EVs RNA sequencing to identify potential miRNA within EVs to illustrate the related mechanisms. There are additional some limitations in our current study: Firstly, our EVs isolation protocol can only preserve small EVs (< 220 nm) by using 0.22 μm filters, however, those larger EVs may also exert unknown effects on BBB and IS recovery. Further studies are required to analyze the biological function of those large EVs in detail. In addition, our isolation procedure by Exo-Prep kit may influence the purity of collected EVs. Although at present there is still no perfect method for achieving EVs with high recovery and specificity, the second-step purification methods (e.g. ultracentrifugation and gradient centrifugation) may be optimal to achieve EVs with high purity and specificity in future studies.

## Conclusions

In summary, we firstly defined high expression of Cav-1 aggravate BBB permeability in early rat pMCAo model, which is related to Cav-1-dependent ZO-1 and Claudin-5 endocytosis. By comparative analysis, our results demonstrated both of BMSC-EVs and BEC-EVs treatments antagonized Cav-1 endocytic pathway for the maintenance of BBB integrity, and the overall therapeutic efficacy of BMSC-EVs was superior to BEC-EVs in acute IS treatment.

## Supplementary Information

**Additional file 1: **Supplemental methods and information of neurological function tests, pcDNA 3.1 vector gene map.

**Additional file 2: Fig. S1.** Characterization of negative control of EVs isolation. (A) Size distribution of negative control sample. (B) Representative low magnificent TEM images of negative control sample. Scale Bar: 1 μm. Magnification: 4k ×. (C) Representative confocal images of b. End3 cells uptake DiI labeled negative control sample. Scale Bar: 20 μm. Magnification: 63 × with 2.5 × zoom in. (D) Representative confocal images and fluorescence quantification of DiI labeled BEC and BMSC-EVs phagocytized by b. End3 cells in different ambient temperatures (n=3). ****P<0.0001, ***P<0.001 vs. 4 ℃ group. Scale Bar: 20 μm. Magnification: 63 × with 2.5 × zoom in.

**Additional file 3: Fig. S2.** Characterization of isolated BMV. (A) Representative western blotting of NeuN, CD 31 in BMV. (B) Representative immunofluorescence staining images of CD 31 in BMV. Scale Bar: 200 μm. Magnification: 10 ×.

**Additional file 4: Fig. S3.** Verification for successful transfection of siRNA and pcDNA 3.1. (A) Representative western blotting and quantification of Cav-1 in b. End3 cells transfected with Ctrl/Cav-1 siRNA and Cav-1 DNA/ pcDNA 3.1 (n=3). ^***^P<0.001, ^**^P<0.01 vs. Ctrl-siRNA/pcDNA 3.1 group. (B) Representative fluorescent microscope images of b. End3 transfected with FAM-negative control siRNA and pcDNA 3.1-GFP. Scale Bar: 200 μm. Magnification: 10 ×.

## Data Availability

All datasets to support current study are available from the corresponding author on reasonable request.

## Abbreviations

BBB: Blood–brain barrier
BMV: Brain microvessel
BEC: Brain endothelial cell
BMSC: Bone marrow mesenchymal stem cell
EB: Evans Blue
EV: Extracellular vesicle
IS: Ischemic stroke
IR: Ischemia and reperfusion
MMP: Matrix metallopeptidase
NTA: Nanoparticle tracking analysis
NVU: Neurovascular unit
OGD: Oxygen and glucose deprivation
PFA: Paraformaldehyde
pMCAo: Permanent middle cerebral artery occlusion
t-PA: Tissue plasminogen activator
TJ: Tight junction
TEM: Transmission electron microscope
