# Supplementary material for: Comparative study of extracellular vesicles derived from mesenchymal stem cells and brain endothelial cells attenuating blood–brain barrier permeability via regulating Caveolin-1-dependent ZO-1 and Claudin-5 endocytosis in acute ischemic stroke
Source: J Nanobiotechnology. 2023 Feb 28;21:70. doi: 10.1186/s12951-023-01828-z (PMC9976550; doi:10.1186/s12951-023-01828-z)

**Comparative study of extracellular vesicles derived from mesenchymal stem cells and brain endothelial cells attenuating blood-brain barrier permeability via regulating Caveolin-1-dependent ZO-1 and Claudin-5 endocytosis in acute ischemic stroke**

**Additional file**

**mNSS and Bederson scores**

For neurological function assessment, 10 rats were included each group randomly via random digits. The higher score the rat was rated under the two rating systems, the severer neurological function deficit it gained after IS. All scoring procedures were conducted by investigators who were blinded to the experiment grouping protocols.

mNSS score scale: 18 points score scale refer to the previous work by J Chen et al (PMID: 11283404):

1. Motor tests (normal: 0; maximum: 3):

Tail raising

1: Flexion of forelimb

1: Flexion of hindlimb

1: Head position alteration >10° to vertical axis in 30 s

1. Walking tests (normal: 0; maximum: 3)

0: Normal walk

1: Unable to straight walk

2: Side circling

3: Side fall down

1. Sensory tests (normal: 0; maximum: 2)

1: Placing test (visual and tactile)

2: Proprioceptive test (deep sensation, pushing the paw against the table edge to stimulate limb muscles)

1. Beam balance tests (normal: 0; maximum: 6)

0: Balances with steady posture

1: Grasps side of beam

2: Hugs the beam and one limb falls down from the beam

3: Hugs the beam and two limbs fall down from the beam, or spins on beam (>60 s)

4: Attempts to balance on the beam but falls off (>40 s)

5: Attempts to balance on the beam but falls off (>20 s)

6: Falls off: No attempt to balance or hang on to the beam (<20 s)

1. Reflexes absent and abnormal movements (normal: 0; maximum: 4)

1: Pinna reflex (head shake when touching the auditory meatus)

1: Corneal reflex (eye blink when lightly touching the cornea with cotton)

1: Startle reflex (motor response to a brief noise from snapping a clipboard paper)

1: Seizures, myoclonus, myodystony

Maximum points: 18

Bederson score referred to the work by Michael Bieber et al (PMID: 31412755):

0: no neurological deficit

1: forelimb flexion

2: forelimb flexion with decreased lateral push resistance

3: circling movement

4: circling movement and display cranial-caudal axis spinning

5: no movement.

Maximum points: 5

**pcDNA 3.1 vector gene map**

pcDNA 3.1(+)/ pcDNA 3.1-GFP vector map (provided by manufacturer):


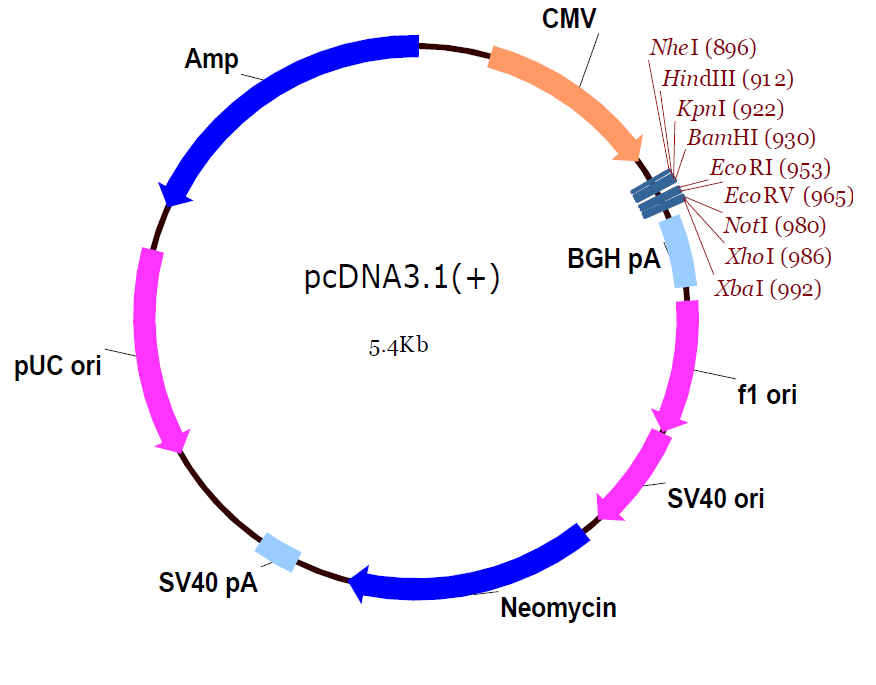


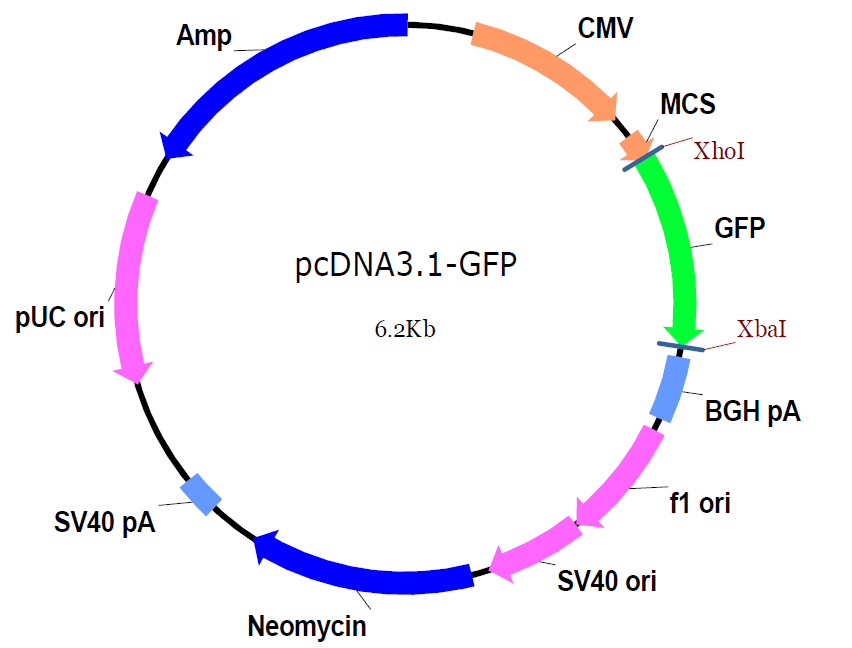

Supplement: Supplementary file 1 — Additional file 1: Supplemental methods and information of neurological function tests, pcDNA 3.1 vector gene map. [file 12951_2023_1828_MOESM1_ESM.docx]
