# Supplementary figures and images for: Comparative study of extracellular vesicles derived from mesenchymal stem cells and brain endothelial cells attenuating blood–brain barrier permeability via regulating Caveolin-1-dependent ZO-1 and Claudin-5 endocytosis in acute ischemic stroke
Source: J Nanobiotechnology. 2023 Feb 28;21:70. doi: 10.1186/s12951-023-01828-z (PMC9976550; doi:10.1186/s12951-023-01828-z)

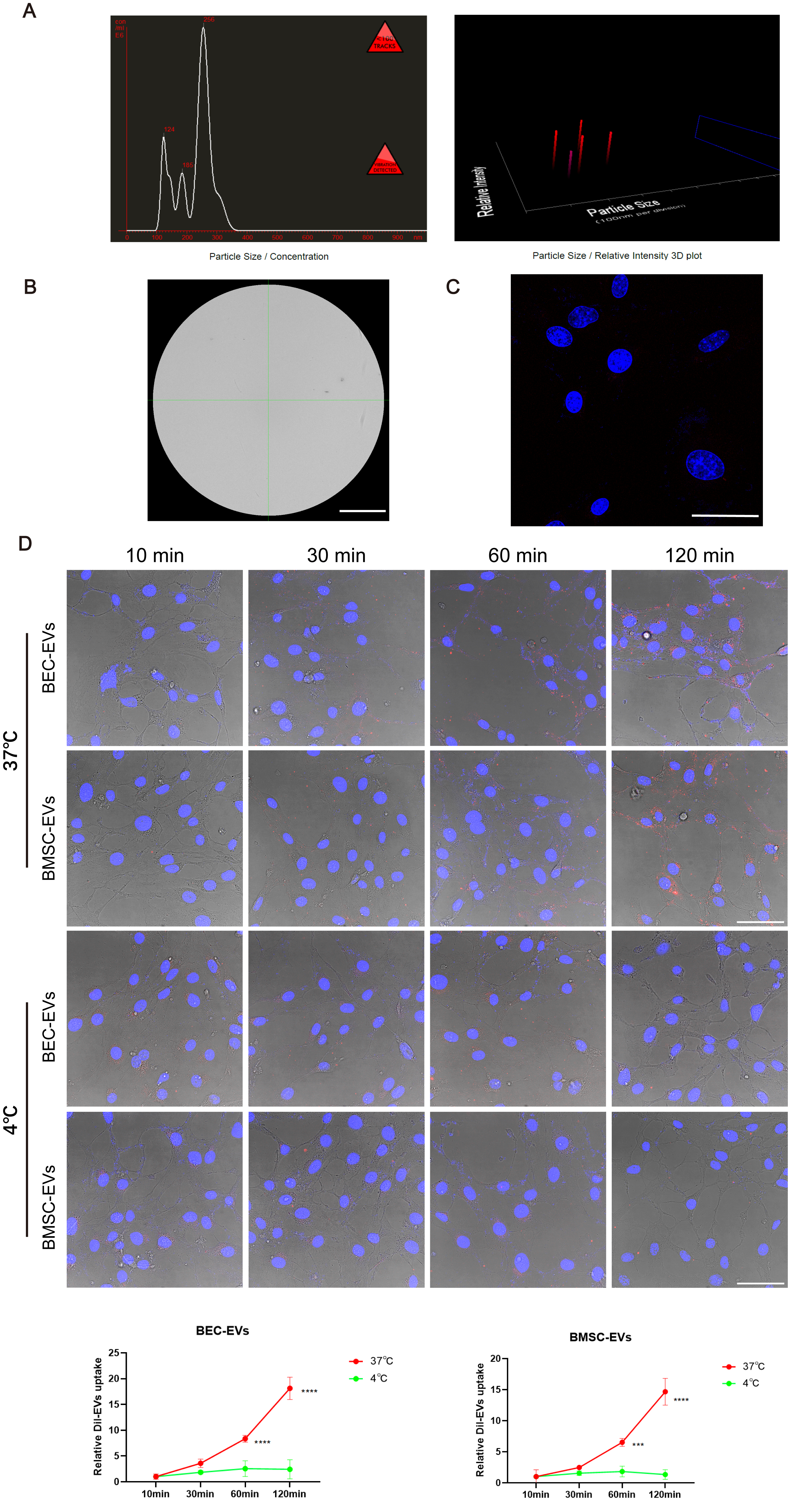

Supplement: Supplementary file 2 — Additional file 2: Fig. S1. Characterization of negative control of EVs isolation. (A) Size distribution of negative control sample. (B) Representative low magnificent TEM images of negative control sample. Scale Bar: 1 μm. Magnification: 4k ×. (C) Representative confocal images of b. End3 cells uptake DiI labeled negative control sample. Scale Bar: 20 μm. Magnification: 63 × with 2.5 × zoom in. (D) Representative confocal images and fluorescence quantification of DiI labeled BEC and BMSC-EVs phagocytized by b. End3 cells in different ambient temperatures (n=3). ****P<0.0001, ***P<0.001 vs. 4 ℃ group. Scale Bar: 20 μm. Magnification: 63 × with 2.5 × zoom in. [file 12951_2023_1828_MOESM2_ESM.tif]

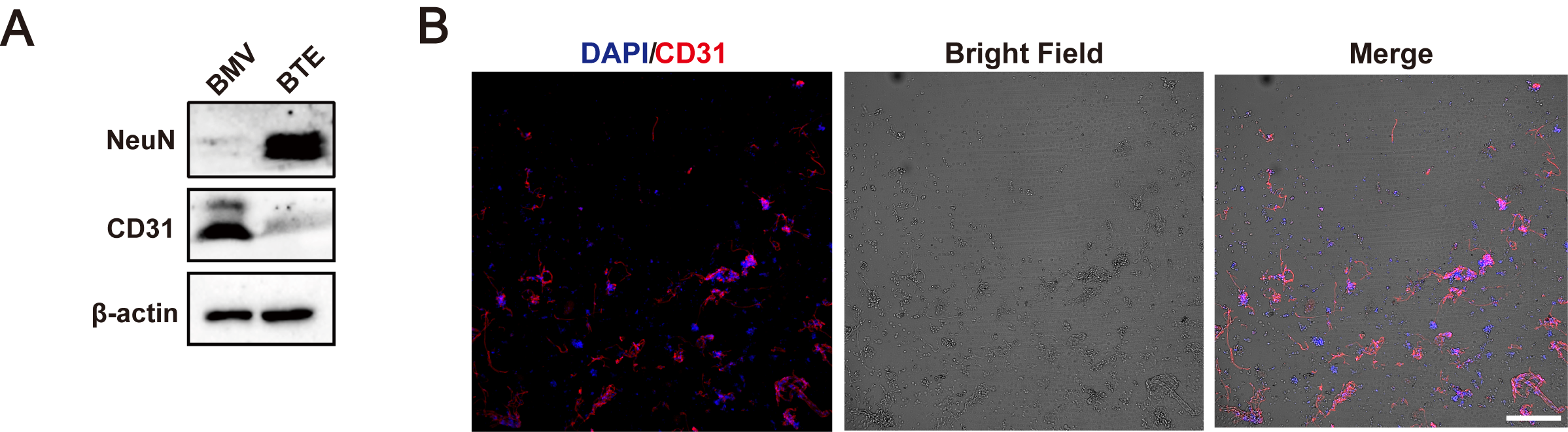

Supplement: Supplementary file 3 — Additional file 3: Fig. S2. Characterization of isolated BMV. (A) Representative western blotting of NeuN, CD 31 in BMV. (B) Representative immunofluorescence staining images of CD 31 in BMV. Scale Bar: 200 μm. Magnification: 10 ×. [file 12951_2023_1828_MOESM3_ESM.tif]

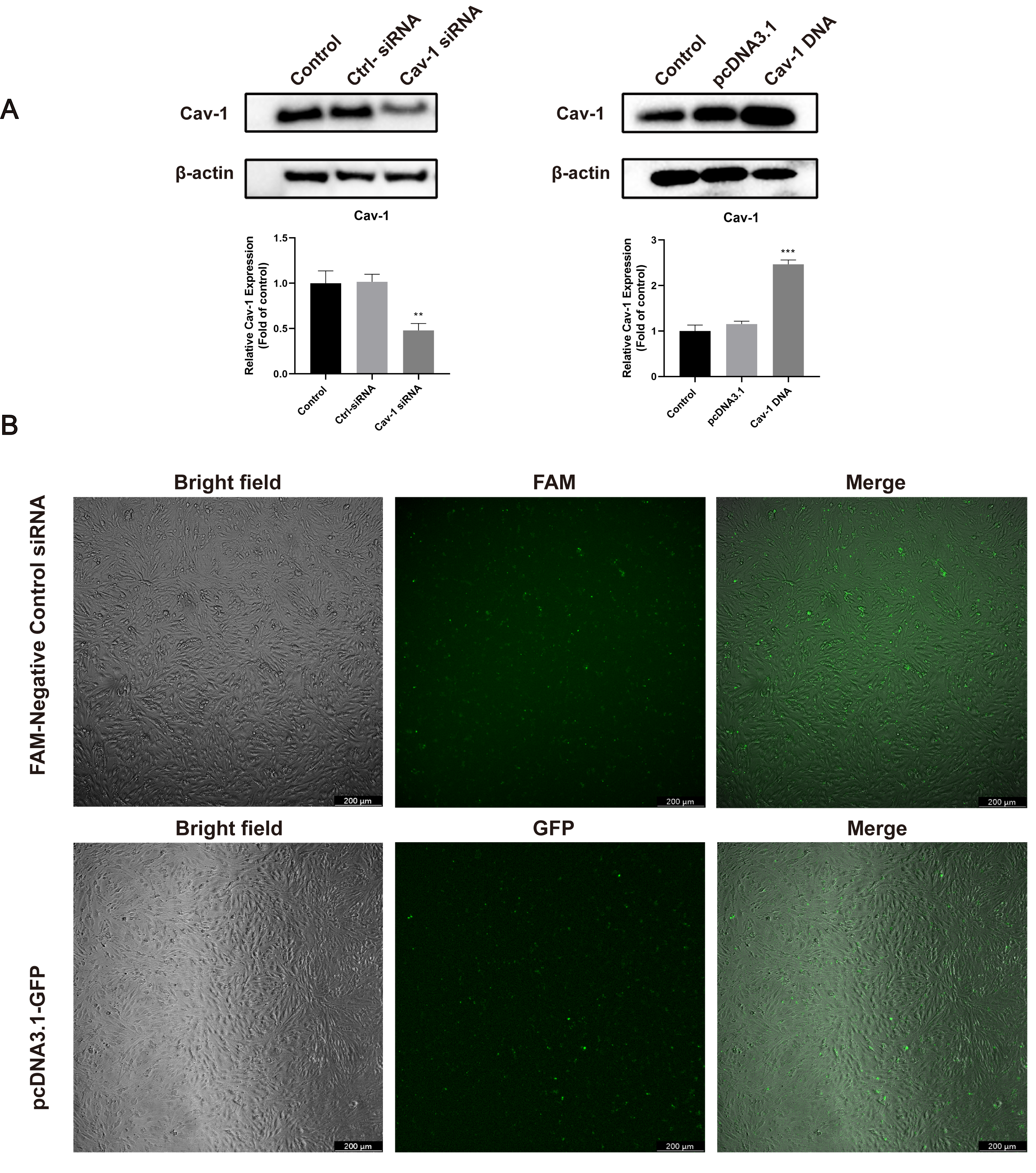

Supplement: Supplementary file 4 — Additional file 4: Fig. S3. Verification for successful transfection of siRNA and pcDNA 3.1. (A) Representative western blotting and quantification of Cav-1 in b. End3 cells transfected with Ctrl/Cav-1 siRNA and Cav-1 DNA/ pcDNA 3.1 (n=3). ***P<0.001, **P<0.01 vs. Ctrl-siRNA/pcDNA 3.1 group. (B) Representative fluorescent microscope images of b. End3 transfected with FAM-negative control siRNA and pcDNA 3.1-GFP. Scale Bar: 200 μm. Magnification: 10 ×. [file 12951_2023_1828_MOESM4_ESM.tif]
